# Risk factor paradox in the occurrence of cardiac arrest in acute coronary syndrome patients

**DOI:** 10.5935/0103-507X.20160065

**Published:** 2016

**Authors:** Silvia Aguiar Rosa, Ana Teresa Timóteo, Marta Afonso Nogueira, Adriana Belo, Rui Cruz Ferreira

**Affiliations:** 1 Hospital de Santa Marta - Lisboa, Portugal.; 2 Sociedade Portuguesa de Cardiologia - Coimbra, Portugal.

**Keywords:** Cardiac arrest, Risk factors, Acute coronary syndrome, Parada cardíaca, Fatores de risco, Síndrome coronariana aguda

## Abstract

**Objective:**

To compare patients without previously diagnosed cardiovascular risk factors) and patients with one or more risk factors admitted with acute coronary syndrome.

**Methods:**

This was a retrospective analysis of patients admitted with first episode of acute coronary syndrome without previous heart disease, who were included in a national acute coronary syndrome registry. The patients were divided according to the number of risk factors, as follows: 0 risk factor (G0), 1 or 2 risk factors (G1 - 2) and 3 or more risk factors (G ≥ 3). Comparative analysis was performed between the three groups, and independent predictors of cardiac arrest and death were studied.

**Results:**

A total of 5,518 patients were studied, of which 72.2% were male and the mean age was 64 ± 14 years. G0 had a greater incidence of ST-segment elevation myocardial infarction, with the left anterior descending artery being the most frequently involved vessel, and a lower prevalence of multivessel disease. Even though G0 had a lower Killip class (96% in Killip I; p < 0.001) and higher ejection fraction (G0 56 ± 10% *versus* G1 - 2 and G ≥ 3 53 ± 12%; p = 0.024) on admission, there was a significant higher incidence of cardiac arrest. Multivariate analysis identified the absence of risk factors as an independent predictor of cardiac arrest (OR 2.78; p = 0.019). Hospital mortality was slightly higher in G0, although this difference was not significant. By Cox regression analysis, the number of risk factors was found not to be associated with mortality. Predictors of death at 1 year follow up included age (OR 1.05; p < 0.001), ST-segment elevation myocardial infarction (OR 1.94; p = 0.003) and ejection fraction < 50% (OR 2.34; p < 0.001).

**Conclusion:**

Even though the group without risk factors was composed of younger patients with fewer comorbidities, better left ventricular function and less extensive coronary disease, the absence of risk factors was an independent predictor of cardiac arrest.

## Abstract (pt)

**Objetivo:**

Comparar pacientes admitidos com síndrome coronariana aguda sem prévia identificação de fatores de risco cardiovascular com pacientes que portavam um ou mais fatores de risco.

**Métodos:**

Análise retrospectiva dos pacientes admitidos com o primeiro episódio de síndrome coronariana aguda sem cardiopatia prévia, incluídos em um registro nacional de síndrome coronariana aguda. Os pacientes foram divididos segundo o número de fatores de risco: nenhum fator de risco (G0), um ou dois fatores de risco (G1 - 2) e três ou mais fatores de risco (G ≥ 3). Realizou-se uma análise comparativa entre os três grupos e se estudaram os preditores independentes de parada cardíaca e óbito.

**Resultados:**

O total apurado foi de 5.518 pacientes, 72,2% deles do sexo masculino, com média de idade de 64 ± 14 anos. O G0 teve uma incidência maior de infarto do miocárdio com elevação do segmento ST, sendo o vaso mais frequentemente envolvido a artéria descendente anterior esquerda, e menor prevalência de envolvimento de múltiplos vasos. Embora o G0 tivesse uma classe Killip mais baixa (96% Killip I; p < 0,001) e maior fração de ejeção (G0: 56 ± 10% *versus* G1 - 2 e G ≥ 3: 53 ± 12%; p = 0,024) na admissão, houve incidência significantemente maior de parada cardíaca. A análise multivariada identificou ausência de fatores de risco como um fator independente para parada cardíaca (OR 2,78; p = 0,019). A mortalidade hospitalar foi ligeiramente maior no G0, embora sem significância estatística. Segundo a análise de regressão de Cox, o número de fatores de risco não se associou com mortalidade. Os preditores de óbito em 1 ano de seguimento foram infarto do miocárdio com elevação do segmento ST (OR 1,05; p < 0,001) e fração de ejeção inferior a 50% (OR 2,34; p < 0,001).

**Conclusão:**

Embora o grupo sem fatores de risco fosse composto de pacientes mais jovens e com menos comorbidades, melhor função ventricular esquerda e coronariopatia menos extensa, a ausência de fatores de risco foi um preditor independente de parada cardíaca.

## INTRODUCTION

Cardiovascular disease is the most important cause of premature death in western societies, and coronary heart disease the leading cause of death worldwide, according to World Health Organization.^(1)^

The main cardiovascular risk factors are well validated, and include, in particular, age, hypertension, diabetes, dyslipidemia, smoking and family history.^(2,3)^ These risk factors are incorporated in cardiovascular risk scores, which are useful tools in clinical practices for stratifying a patient's risk of coronary artery disease and cardiovascular death and to guide the diagnosis and treatment approach.^(3-5)^

However, among patients admitted with acute coronary syndrome (ACS), there is a subgroup whose pre-event stratification classifies them as low cardiovascular risk, due to the absence of traditional risk factors.^(6)^

Limited data are available regarding the magnitude, clinical features and outcome of ACS in individuals without risk factors.

The aim of the present study is to analyze the baseline characteristics, clinical presentation, laboratory, echocardiographic and angiographic characteristics and outcome of patients without previously diagnosed risk factors who were admitted with a first episode of ACS. With regards to hospital outcome, the presence of heart failure, cardiogenic shock and cardiac arrest was analyzed. In hospital and one-year follow up mortality was also evaluated, and was designated as the primary endpoint. The presence of cardiac arrest was considered as the secondary endpoint. The authors performed a comparison between groups according to the number of risk factors.

## METHODS

This study was a retrospective analysis of patients admitted with first episode of ACS without previous heart disease, who were included in the National Portuguese ACS registry (Pro ACS) in each of the 33 participant cardiology departments, between 2010 and 2014. The Portuguese Registry of ACS received the approval and authorization from the National Committee of Data Protection (authorization number 3140/2010), and is registered at ClinicalTrials.gov with the identification number, NCT 01642329. An informed consent form was also given to all patients. Patients who presented symptoms thought to be due to ACS and electrocardiographic changes consistent with and/or elevated levels of biomarkers of myocardial necrosis were included in the registry. This study includes patients with ST-segment elevation myocardial infarction (STEMI), non-ST-segment elevation myocardial infarction (NSTEMI) and unstable angina. STEMI was defined as a persistent ST segment elevation for more than 30 minutes, and the remaining cases were considered non-ST-elevation ACS, NSTEMI, if their troponin level was elevated above the reference limit, and unstable angina, if there were no changes in biomarkers. The diagnosis was defined by the physician at hospital admission.

The patients were divided into 3 groups, according to the number of risk factors, as follows: 0 risk factor (G0), 1 or 2 risk factors (G1 - 2) and 3 or more risk factors (G ≥ 3). The following risk factors were analyzed: age > 55 years in men and > 65 years in women, hypertension, diabetes, dyslipidemia, smoking, family history of coronary artery disease. The presence of risk factors was based on the patients' medical history.

In each patient, baseline clinical characteristics, including demographic characteristics and comorbidities, were collected. Laboratory data on admission, electrocardiographic and echocardiographic parameters were also analyzed.

The outcome variables studied were cardiac arrest (at the prehospital level or in-hospital) and in-hospital and one-year all cause mortality.

The study protocol is in accordance with the Declaration of Helsinki.

### Statistical analysis

Statistical analysis was performed using dedicated software, Statistical Package for Social Sciences (IBM SPSS, Chicago, IL), v. 19. Continuous variables were expressed as the mean ± standard deviation, and categorical variable were expressed as percentages. Study groups were compared using ANOVA for continuous variables, and Pearson's chi-square test for categorical measures.

Two multivariate logistic regression models were built to identify the predictors of two endpoints, cardiac arrest and hospital mortality. To perform each regression model, we considered the variables that were significantly associated with the endpoint (p < 0.100 at univariate analysis) and had clinical relevance. The variables that were included in the final model were selected by the Stepwise Forward method, considering Likelihood Ratio test. The estimated odds ratio was considered to assess risk. Since we considered some variables as continuous, the linearity of logit for each variable was tested by the method of fractional polynomials. Goodness of fit was evaluated by model calibration and classification accuracy. To test the model calibration, the Hosmer and Lemeshow test (HL) was used, and classification accuracy was assessed by area under the ROC curve (AUC) analysis.

The predictors of death at one-year follow up were determined by Cox regression model. Once again, we considered variables that were significantly associated with the endpoint and had clinical relevance, and used the Stepwise Forward method considering Likelihood Ratio test to select variables. The estimated hazard ratio was considered to assess risk. The proportionality of the risks war assessed by analyzing the Schoenfeld residuals, and the functional form of a continuous variable was analyzed considering Martingale residuals.

95% confidence intervals (CI) were used, and a p-value < 0.05 was considered statistically significant.

## RESULTS

During the study period, 5,518 patients were admitted with a first episode of ACS and with no previous heart disease (49.7% of all patients enrolled in ProACS registry in the same period), and were included in this analysis.

**Figure 1:**
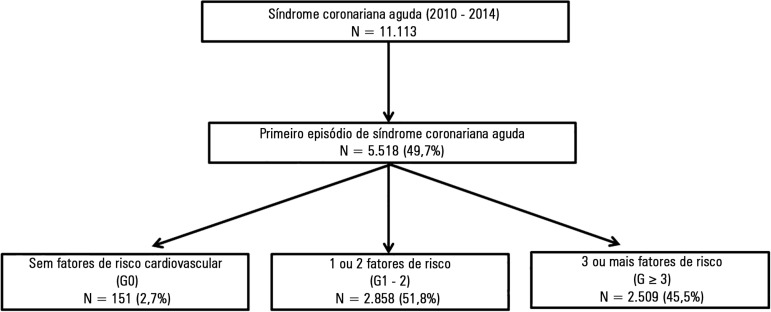
Study flowchart.

The majority of patients were male (72.2%), with a mean age of 64 ± 14 years. In total, 151 patients (2.7%) were included in G0, 2,858 (51.8%) in G1 - 2 and 2,509 (45.5%) in G ≥ 3 (Figure 1).

The baseline characteristics of the three groups are presented in table 1. Patients in G0 were significantly younger, with lower ratio male/female when comparing with G1-2 and G ≥ 3. Patients without risk factors also presented significantly fewer comorbidities, particularly peripheral arterial disease, previous stroke and chronic kidney disease.

**Table 1:** Baseline clinical characteristics

Characteristics	G0 (N = 151)	G1 - 2 (N = 2,858)	G ≥ 3 (N = 2,509)	p value
Male	64.20	73.50	71.10	0.014*
Age (years)	49 ± 8	62 ± 15	67 ± 12	< 0.001†
Body mass index (kg/m^2^)	26.7 ± 3.9	26.8 ± 4.2	27.8 ± 4.3	< 0.001†
Hypertension	0	40.6	88.2	< 0.001*
Diabetes	0	6.0	44.1	< 0.001*
Dyslipidemia	0	23.7	77.9	< 0.001*
Smoker	0	34.8	34.4	< 0.001*
Family history of coronary artery disease	0	4.5	11.0	< 0.001*
Peripheral arterial disease	0.7	1.8	3.9	< 0.001*
Previous stroke	1.3	4.2	9.2	< 0.001*
Chronic kidney disease	2.7	2.5	5.4	< 0.001*
Neoplasm	3.4	4.2	4.4	0.855*
Chronic obstructive pulmonary disease	2.1	3.8	4.8	0.079*

Chronic kidney disease: creatinine > 2.0mg/dL, hemodialysis or renal transplantation.

* Chi-squared test;

† ANOVA. Values are expressed as (%) and mean ± standard deviation.

During acute events, an extensive blood analysis was performed. In G0, 7.1% of patients presented with admission blood glucose higher than 200mg/L, and 13.0% of patients had total cholesterol higher than 240mg/dL.

Regarding ACS clinical presentation (Table 2), G0 had a greater incidence of STEMI, but lower Killip class, heart rate and systolic blood pressure on admission.

**Table 2:** Admission characteristics and hospital outcome

Characteristics	G0 (N = 151)	G1 - 2 (N = 2,858)	G ≥ 3 (N = 2,509)	p value
Angina	95.4	92.1	90.6	0.027*
Dyspneia	0	2.5	3.7	0.004*
Syncope	2.0	2.2	2.3	0.966†
STEMI	57.6	54.3	47.3	< 0.001*
Killip class I	96.0	90.2	86.5	< 0.001*
Heart rate (bpm)	76 ± 18	76 ± 19	79 ± 19	< 0.001†
Sinus rhythm	95.4	93.2	91.5	0.028*
Systolic blood pressure (mmHg)	132 ± 22	136 ± 28	141 ± 31	< 0.001†
Hemoglobin on admission (g/dL)	14 ± 1.6	14 ± 1.8	13.8 ± 1.9	< 0.001†
Left ventricular ejection fraction	56 ± 10	53 ± 12	53 ± 12	0.024†
Culprit artery - left anterior descending artery	41.0	40.6	36.6	0.027*
Multivessel disease	16.3	39.7	51.5	< 0.001*
Percutaneous coronary intervention	70.0	72.7	70.4	0.164*
Heart failure	4.0	11.6	13.1	0.002*
Cardiogenic shock	1.4	3.5	3.6	0.355*
Cardiac arrest	6.6	3.0	2.7	0.021*
Mortality	4.0	3.4	3.5	0.917*

STEMI - ST segment elevation myocardial infarction;

* Chi-squared test;

† ANOVA. Values are expressed as (%) and mean ± standard deviation.

Comparing G1 - 2 and G ≥ 3, echocardiography documented significantly less left ventricular systolic function impairment in G0, with a mean ejection fraction of 56 ± 10%. This fact is likely related to the lower incidence of heart failure during hospitalization in this group (Table 2).

The left anterior descending artery was the most frequently involved vessel in G0 patients, despite these individuals presenting with a lower incidence of multivessel coronary disease, compared with known risk factors patients. There was no significant difference in percutaneous coronary intervention between the three groups (Table 2).

During hospitalization, G0 patients presented a twofold higher incidence of cardiac arrest, when compared with the G1 - 2 and G3 groups (6.6% *versus* 3.0% *versus* 2.7%; p = 0.021). However, G0 patients did not have a significantly higher hospital mortality (Table 2).

A logistic regression model was built to identify the predictors of cardiac arrest, including the absence of risk factors, STEMI, systolic blood pressure, heart rate, Killip class > I, creatinine at admission, previous and in-hospital medication, culprit artery (left main and left anterior descending artery), percutaneous coronary intervention and left ventricular ejection fraction < 50%. This analysis identified the absence of risk factors as an independent predictor of cardiac arrest (OR = 2.78; 95%CI 1.19 - 6.51; p = 0.019). The other independent predictors were STEMI (OR = 5.74; 95%CI 3.18 - 10.38; p < 0.001), higher heart rate (OR = 1.02; 95%CI 1.01 - 1.02; p < 0.001), systolic blood pressure (OR = 0.99; 95%CI 0.98 - 0.99; p < 0.001), Killip class > I (OR = 3.55; 95%CI 2.27 - 5.56; p < 0.001) and nitrates administration during hospitalization (OR = 0.53; 95%CI 0.34 - 0.83; p = 0.005). The model was well calibrated (HL: p = 0.097), and had good discriminant accuracy (AUC = 0.79; 95%CI 0.76 - 0.82) (Table 3).

**Table 3:** Statistical analysis to determine the predictors of cardiac arrest

Variables			Multivariate analysis		Univariate analysis	
	Coefficient	SE	p value*	OR (95%CI)	p value*	OR (95%CI)
Risk factor 0†	1.022	0.434	0.019	2.78 (1.19 - 6.51)	0.007	2.57 (1.30 - 5.11)
Risk factors 1 - 2†	0.126	0.200	0.529	1.13 (0.77 - 1.68)	0.511	1.12 (0.81 - 1.54)
STEMI	1.748	0.302	< 0.001	5.74 (3.18 - 10.38)	< 0.001	6.32 (4.02 - 9.94)
Heart rate	0.016	0.004	< 0.001	1.02 (1.01 - 1.02)	< 0.001	1.01 (1.01 - 1.02)
SBP	-0.013	0.003	< 0.001	0.99 (0.98 - 0.99)	< 0.001	0.98 (0.97 - 0.98)
KK > 1	1.266	0.229	< 0.001	3.55 (2.27 - 5.56)	< 0.001	4.17 (2.97 - 5.87)
Nitrates_in-hospital_	-0.634	0.227	0.005	0.53 (0.34 - 0.83)	< 0.001	0.43 (0.30 - 0.61)

SE - standard error; OR - odds ratio; 95%CI - 95% confidence intervals; STEMI - ST segment elevation myocardial infarction; SBP - systolic blood pressure; KK - Killip Kimball class.

* Wald test;

† comparing with 3 or more risk factors.

Hospital all-cause mortality was slightly higher in G0, although this difference was not significant (Table 2). By logistic regression, we conclude that the absence of risk factors was not an independent predictor of hospital mortality (OR = 2.37; 95%CI 0.30 - 18.76; p = 0.414). Independent predictors included STEMI (OR = 2.75; 95%CI 1.73 - 4.38; p < 0.001), Killip class > I (OR = 2.19; 95%CI 1.43 - 3.34; p < 0.001), no percutaneous coronary intervention (OR = 4.90; 95%CI 3.08 - 7.80; p < 0.001) and left ventricular ejection fraction < 50% (OR = 3.72; 95%CI 2.36 - 5.87; p < 0.001). The model was well calibrated (HL: p = 0.147), and had excellent discriminant accuracy AUC = 0.92; 95%CI 0.89 - 0.94) (Table 4).

**Table 4:** Statistical analysis to determine the predictors of hospital mortality

Variables			Multivariate analysis		Univariate analysis	
	Coefficient	SE	p value*	OR (95%CI)	p value*	OR (95%CI)
Risk factor 0†	0.862	1.056	0.414	2.37 (0.30; 18.76)	0.764	1.14 (0.49 - 2.65)
Risk factors 1 - 2†	0.028	0.204	0.892	1.03 (0.69; 1.53)	0.818	0.97 (0.72 - 1.30)
Age	0.068	0.009	< 0.001	1.07 (1.05; 1.09)	< 0.001	1.10 (1.08 - 1.11)
STEMI	1.011	0.237	< 0.001	2.75 (1.73; 4.38)	< 0.001	3.11 (2.22 - 4.35)
SBP	-0.017	0.004	< 0.001	0.98 (0.98; 0.99)	< 0.001	0.97 (0.96 - 0.97)
KK > 1	0.783	0.216	< 0.001	2.19 (1.43; 3.34)	< 0.001	8.54 (6.32 - 11.53)
Beta-blocker_in-hospital_	-0.927	0.225	< 0.001	0.40 (0.25; 0.61)	< 0.001	0.13 (0.10 - 0.18)
ACEI/ARB_in-hospital_	-0.922	0.240	< 0.001	0.40 (0.25; 0.64)	< 0.001	0.12 (0.09 - 0.16)
No Cor/No PCI‡	1.590	0.237	< 0.001	4.90 (3.08; 7.80)	< 0.001	8.96 (6.52 - 12.29)
Cor/No PCI‡	0.509	0.326	0.119	1.66 (0.88; 3.15)	0.830	0.83 (0.51 - 1.36)
LVEF < 50%	1.314	0.233	< 0.001	3.72 (2.36; 5.87)	< 0.001	7.14 (4.75 - 10.71)

SE - standard error. OR - odds ratio; 95%CI - 95% confidence intervals; STEMI - ST Segment elevation myocardial infarction; SBP - systolic blood pressure; KK - Killip-Kimball class; BB - beta-blocker; ACEI/ARB - angiotensin converting enzyme inhibitors/angiotensin II receptor blockers; Cor - coronary angiography; PCI - percutaneous coronary intervention, LVEF - left ventricular ejection fraction.

* Wald test;

† comparing with 3 or more risk factors;

‡ comparing to coronary angiography/percutaneous coronary intervention.

**Figure 2:**
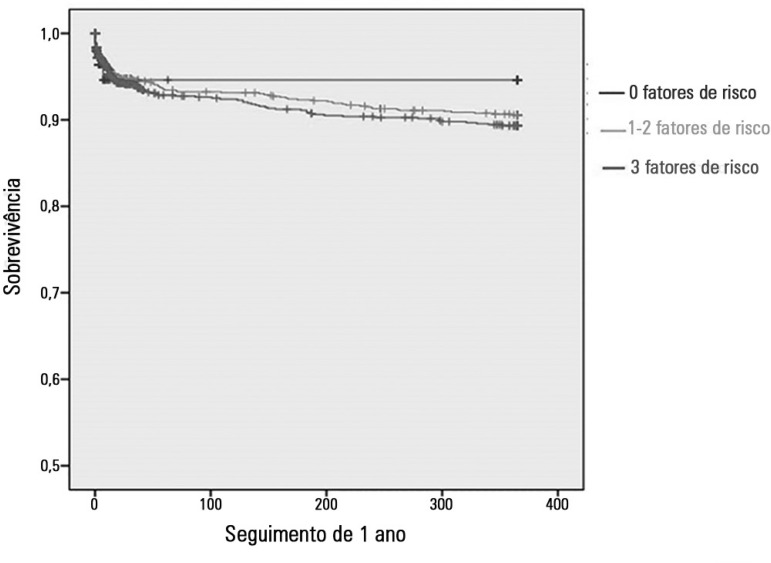
Kaplan Meier survival curves for the three study groups.

At the one-year follow up, there was no significant difference in survival between the three groups (Figure 2). By Cox regression analysis, the number of risk factor was not found to be associated with mortality (HR = 0.78; 95%CI 0.45 - 1.37; p = 0.393). The predictors of death at the one-year follow up were as follows: age (HR = 1.05; 95%CI 1.03 - 1.06; p < 0.001), STEMI (HR = 1.94; 95%CI 1.25 - 3.02; p = 0.003) and ejection fraction < 50% (HR = 2.34; 95%CI 1.57 - 3.47; p < 0.001) (Table 5).

**Table 5:** Statistical analysis to determine predictors of death at the one-year follow up

Variables			Multivariate analysis		Univariate analysis	
	Coefficient	SE	p value*	OR (95%CI)	p valor*	OR (95%CI)
Risk factor 0 - 1†	0.244	0.286	0.393	0.78 (0.45; 1.37)	0.173	0.81 (0.59 - 1.10)
Age	0.046	0.009	< 0.001	1.05 (1.03; 1.06)	< 0.001	1.09 (1.08 - 1.10)
STEMI	0.664	0.225	0.003	1.94 (1.25; 3.02)	< 0.001	2.33 (1.80 - 3.01)
ACEI/ARB_discharge_	-0.598	0.227	0.008	0.55 (0.35; 0.86)	< 0.001	0.18 (0.13 - 0.25)
BB_discharge_	-0.851	0.221	< 0.001	0.43 (0.28; 0.66)	< 0.001	0.16 (0.11 - 0.22)
ASA_discharge_	-1.460	0.229	< 0.001	0.23 (0.15; 0.36)	< 0.001	0.08 (0.06 - 0.11)
No Cor/No PCI‡	0.784	0.248	0.002	2.19 (1.35; 3.56)	< 0.001	6.95 (5.41 - 8.92)
Cor/No PCI‡	-0.251	0.323	0.439	0.78 (0.41; 1.47)	0.838	0.96 (0.67 - 1.38)
LVEF<50%	0.848	0.202	< 0.001	2.34 (1.57; 3.47)	< 0.001	4.55 (3.44 - 6.02)

SE - standard error; OR - odds ratio; 95%CI - 95% confidence intervals; RF- risk factors; STEMI - ST Segment elevation myocardial infarction; ACEI/ARB - angiotensin converting enzyme inhibitors/Angiotensin II receptor blockers; BB - beta-blocker; ASA - acetylsalicylic acid; Cor - coronary angiography; PCI - percutaneous coronary intervention, LVEF - left ventricular ejection fraction.

* Wald test;

† comparing with 3 or more risk factors;

‡ comparing to coronary angiography/percutaneous coronary intervention.

## DISCUSSION

In the ProACS registry, patients with no known risk factors previous to the index event represent less than 3% of the overall ACS population without previous coronary artery disease. This proportion is in line with previous published data, which also showed that about 2% of patients admitted with a first episode of ACS had no risk factor.^(6)^ Surprisingly, in this study, the absence of risk factors was associated with higher incidence of cardiac arrest.

In our population, patients without known risk factors were younger, had less comorbidities and better left ventricular systolic function. Even though this group of patients had less multivessel disease, they presented more often with STEMI and more frequently had the left anterior descending artery as the culprit. This fact has been described previously in other national registries, in which younger patients had higher STEMI incidence.^(7,8)^ Our findings are in accordance with previous studies that showed a higher incidence of single-vessel disease in these patients.^(9-11)^

In our registry, the absence of risk factors was an independent predictor of cardiac arrest on presentation and hospitalization. However, hospital mortality was not significantly higher in G0 patients. Previous studies showed an inverse relationship between number of risk factors and hospital mortality. However, in a study by Canto et al., patients without risk factors were older, had more cardiogenic shock and higher Killip class, which is a different population from that in our registry.^(12)^ Also, in a CRUSADE sub-study, an inverse association between number of risk factors and mortality was reported in the non-ST-segment elevation myocardial infarction population.^(13)^

We can postulate that patients with more risk factors and higher frequency of multivessel disease have more collateral blood flow, and this fact can limit infarct size and consequently, reduce hospital mortality and cardiac arrest. On the other hand, in the absence of risk factors, an ACS is less likely, and a lower suspicion can delay the diagnosis and effective intervention, increasing the risk of ventricular arrhythmia and mortality.

In contrast to hospital outcome, the one-year survival was higher in patients without risk factors. This fact likely reflects the younger age, better left ventricular function and fewer comorbidities of these patients.

Some of the patients without known risk factors might have another less conventional RF that was not assessed, since other risk factors is not systematically collected in the ProACS registry.

Patients without traditional risk factors can have, however, changes in glycemic metabolism, such as prediabetes and insulin resistance, which are correlated with the atherogenic process. This group of patients may have a sedentary lifestyle, with physical inactivity and/or poor nutrition and abdominal obesity that can contribute to disease progression. Depression was also previously described as a risk factor for ACS.^(14,15)^ These patients can also have atypical etiology, with hereditary thrombophilia and hyperhomocysteinemia being the most frequent etiologies described in previous studies.^(9,16)^

Little is known about the physiopathology of ACS in patients without traditional risk factors, and more studies are needed to understand these events and their correlation with poor hospital outcome.

Our study, based on a national registry with a large number of patients and recent data, accurately reflects clinical practice. Since the data was drawn from a registry, this research study does not have selection bias, and the study population dimension allowed the determination of outcome predictors.

### Study limitations

A registry has the advantage of representing real life clinical practice, and the findings of the study are probably applicable to a large number of tertiary hospitals. However, only traditional risk factors were reported, and as we do not have information regarding other types of risk factors, we cannot conclude which atypical factors might be associated with the worsened outcome observed. Additionally, the diagnoses were performed by different physicians in each department, which could generate some bias. Furthermore, a minority of patients without known risk factors presented evidence of diabetes and dyslipidemia in blood samples collected during the acute event.

Finally, as the registry does not collect detailed information on the cause of death and thus, only the all-cause mortality data was presented.

## CONCLUSION

Even though the group with no risk factors was composed of younger patients with fewer comorbidities, better left ventricular function and less extensive coronary disease, the absence of risk factors was, in this study, an independent predictor of cardiac arrest. Even though patients without risk factors presented with a two times higher incidence of cardiac arrest during hospitalization, the absence of risk factors was not correlated with the occurrence of higher all-cause mortality. It is important to emphasize that despite these patients being less diseased at baseline, their hospital mortality was similar, and as such, these patients required the same effort in treatment approach. Importantly, at the one-year follow up, there was no significant difference in survival between study groups, and patients without risk factors presented a survival rate that was slightly better, reflecting the absence of important comorbidities.
